# Antibiotic use in the care home setting: a retrospective cohort study analysing routine data

**DOI:** 10.1186/s12877-015-0073-5

**Published:** 2015-06-25

**Authors:** Pär-Daniel Sundvall, Beth Stuart, Martin Davis, Paul Roderick, Michael Moore

**Affiliations:** Research and Development Unit, Primary Health Care in Southern Älvsborg County, Sven Eriksonsplatsen 4, SE-503 38 Borås, Sweden; Department of Public Health and Community Medicine, Institute of Medicine, the Sahlgrenska Academy, University of Gothenburg, Box 100, SE-405 30 Gothenburg, Sweden; Academic Unit of Primary Care and Population Sciences, Faculty of Medicine, University of Southampton, Mailpoint 805, C floor, South Academic Block, Southampton General Hospital, Southampton, SO166YD UK

**Keywords:** Anti-bacterial agents, Nursing homes, Homes for the aged, Urinary tract infections, Bacteriuria, Family practice

## Abstract

**Background:**

Point prevalence studies in care homes show a high use of antibiotics, especially to treat urinary tract infections (UTI). There is a lack of large studies presenting annual antibiotic prescription data in care homes compared to those not in care homes. This study aimed to describe the pattern of antibiotic prescribing in those 75 years and over, with a focus on UTI.

**Methods:**

In this retrospective longitudinal cohort study we used the Hampshire Health Record (HHR) containing routine data from general practices in Hampshire area, UK covering 1.24 million residents. Data were extracted throughout 2011 from the Hampshire Health Record on age, gender, care home status, antibiotic prescriptions, urinary catheters and comorbidity. Prescription pattern expressed as rate per 100 people. Nursing home residence defined by postcode. Logistic regression was used to assess independent risk of one or more antibiotic prescriptions in care home residents adjusting for age, sex and comorbidity, separately by catheter use.

**Results:**

102,020 of 1,244,313 residents in the Hampshire Health Record (8.2 %) were aged ≥75 years of whom 7481 (7.3 %) were resident in care home settings. The annual antibiotic prescriptions increased from 53/100 inhabitants among those <75 years, to 142/100 among those ≥75 years not in a care home and to 199/100 among those ≥75 years in a care home. Care home residents with urinary catheters (4.4 %) had even higher use at 440/100 versus 188/100 if no catheter. UTI antibiotics showed a similar but more rapidly increasing pattern. For those in care homes without a urinary catheter, the odds ratio was 2.2 (2.1–2.3) higher for prescriptions of UTI antibiotics compared to those not in care homes after adjusting for age, gender and comorbidity. For those with a urinary catheter the odds ratio was 1.4 (1.1–1.8) for UTI antibiotics compared to those not in care homes. For all antibiotics the odds ratio was 1.2 (1.2–1.3).

**Conclusions:**

Residence in a care home setting is associated with high antibiotic consumption; this is especially evident for UTI antibiotics where the odds of prescription is doubled.

## Background

The increasing rate of antibiotic resistance is a huge problem as modern health care depends on effective antibiotics to treat bacterial infections [1]. Use of antibiotics increases the risk of antibiotic resistance [2–4], thus it is important to promote a rational use of antibiotics. Antibiotic resistant bacteria selected by antibiotic treatment, can also be spread among residents at nursing homes [5].

Patients in care home settings are frequently prescribed antibiotics on an empirical basis for presumed urinary tract infection when suffering from symptoms not specific to the urinary tract [6, 7]. The evidence base for such treatment is poor [8–10] and the high prevalence of asymptomatic bacteriuria adds to the diagnostic difficulty [11]. UTI diagnosis is often made in the absence of newly onset focal urinary tract symptoms [12, 13]. When suspecting UTI in nursing home residents with advanced dementia, the presenting symptom was dysuria in only 3.8 %, urinary frequency in 1.5 % and urinary urgency in 0 % of the episodes [12]. Therefore it is likely that a substantial proportion of these UTI antibiotics are of dubious value. A correct diagnosis is important to avoid a potentially harmful antibiotic treatment and possible delay of other diagnoses.

The nurse plays a central role in the prescription of UTI antibiotics through an awareness of changes in residents’ mental status and their consequential communication of this to physicians [6, 14]. Due to this awareness among nurses, residents of care homes might be prescribed more antibiotics for suspected UTI than elderly individuals at the same age and with the same number of co-morbidities but not living in care homes.

The majority of previous studies of antibiotic use in care homes have not compared prescription rates to elderly not living in care homes. Furthermore, most studies of antibiotic use have been point prevalence studies which cannot evaluate annual prescription rates due to substantial seasonal variation in antibiotic prescriptions [15]. There is a lack of studies evaluating annual antibiotic prescription rates among elderly residents of care homes compared to elderly not living in care homes.

The aim of this study was using routine data to describe the pattern of antibiotic prescribing in those 75 years and over resident in care home settings compared to community dwelling older people, with a focus on urinary tract infections taking account of urinary catheter status.

## Methods

We interrogated routine data in a pseudonymised form from Hampshire primary health care records in the Hampshire Health Record (HHR). The HHR contains routine data from 60 % of the practices in Hampshire in southern England with over one million clinical records. All those in the HHR 1 January 2011 still alive on 31st December 2011 were included in this retrospective longitudinal cohort study and followed up throughout 2011.

### Ethics consideration

Use of the Hampshire Health Record Analytical database (HHR-A) for research is regulated by the Hampshire Health Record Advisory Group (HHRAG) and the NHS South Commissioning Support Unit Business Intelligence team. HHR-A data is pseudonymised and therefore it is not possible to identify patients. These governance mechanisms mean that data from the HHR-A can be examined with HHRAG approval but without the need for formal ethical approval. HHRAG approved our use of HHR-A data for this study (reference number HHRA Request 12424).

### Collecting data to characterise the population

Individuals ≥75 years were characterised by living in care home settings or not. In this paper, care homes consists of residential care homes and nursing homes. In a residential care home in the UK there is staff available 24 h a day and in a nursing home there is also a qualified nurse on duty 24 h a day.

In the HHR, clinical coding associated with care home residency was very low and could not be used in the analysis. As a proxy we extracted data using postcode data and assumed those aged ≥75 years in individual postcodes for nursing homes, 1 January 2011, were care home residents for the year 2011.

Data on number of comorbidities using Quality and Outcomes Framework (QOF) register coding was extracted as well as age and gender. QOF is a system for the performance management of general practitioners in the National Health Service (NHS) in the UK and since this is linked to payment the registers are well maintained. The QOF register in HHR includes, asthma, atrial fibrillation, cancer, chronic heart disease, chronic kidney disease, chronic obstructive pulmonary disease, dementia, depression, diabetes mellitus, epilepsy, heart failure, hypertension, learning disabilities, serious mental health conditions, neurological condition, obesity, palliative care, renal disease, stroke or transient ischaemic attack and vascular disease.

### Prescriptions of antibiotics and urinary catheters

Data on primary care prescriptions of antibiotics were extracted as well as prescriptions of urinary catheters. Antibiotics were categorised according to the British National Formulary. The antiseptic substance methenamine hippurate is not an antibiotic and has no influence on antibiotic resistance. Throughout this paper, methenamine hippurate is consequently excluded whenever antibiotics are referred to or presented.

In the HHR, clinical coding associated with antibiotic prescription was extremely low and could not be used in the analysis. Trimethoprim and nitrofurantoin are the recommended first line treatment for urinary tract infection in the UK (Primary Care Guidance, Public Health England). In an observational study trimethoprim was prescribed for UTI in >75 % of cases [16]. In this study other antibiotics were prescribed but trimethoprim and nitrofurantoin are rarely used for alternative conditions, thus prescription of these two antibiotics was assumed to be for urinary tract infection. Furthermore, fosfomycin was not commonly used in primary care in the UK in 2011.

Those prescribed indwelling urinary catheters on NHS prescription list were classified as urinary catheter users.

### Statistical analysis

The population was described by number of individuals, age, gender, having a urinary catheter or not and care home status for those age ≥75 years. The proportion of individuals prescribed any antibiotics (UTI prophylactic antibiotics included) at least once during 2011 was calculated as well as the mean number of prescriptions per 100 inhabitants. These prescription rates were calculated for any antibiotics as well as for antibiotics divided into UTI antibiotics and non UTI antibiotics.

Above calculations were done for the whole cohort and then for the following subgroups; individuals <75 years, individuals ≥75 years not in care home settings and individuals ≥75 years in care home settings. Those in care home settings were further divided in those with and without urinary catheters.

### Logistic regressions

To assess associations and predictors of antibiotic prescriptions among those ≥75 years, univariate analysis and logistic regressions were performed. In these analyses we aimed to describe antibiotic treatment of infections, not antibiotic prophylaxis. In the regression analyses we thus excluded patients receiving six or more prescriptions of nitrofurantoin 50 mg and/or trimethoprim 100 mg in 2011 as these patients were assumed to be prescribed long-term prophylaxis against recurrent UTI rather than being treated for individual UTI episodes.

In those ≥75 years, not assumed to be prescribed long-term prophylaxis against recurrent UTI, a series of univariate analyses were performed with the following independent variables; age, gender, being a care home resident or not and number of comorbidities. Two different series were performed, first with the dependent variable “being prescribed any antibiotics at all during 2011” and then with the dependent variable “being prescribed at least one UTI antibiotic (nitrofurantoin or trimethoprim) during 2011”.

Significant factors from the univariate analysis were included in a logistic regression model to assess the association between being a care home resident and prescriptions of any antibiotics and UTI antibiotics respectively, adjusting for covariates. Forward selection was used; variables were included if significant at the 10 % level and retained in multivariate analysis if they remained significant at the 5 % level. We tested for an interaction between catheter status and care home status in order to determine whether there was evidence of a differential effect on antibiotic prescribing of being in a care home by catheter status. Where there was evidence of a significant interaction, the calculations were performed separately for the catheter and non-catheter groups.

Stata version 12.1 (StataCorp) was used for the statistical analysis.

## Results

**Fig. 1:**
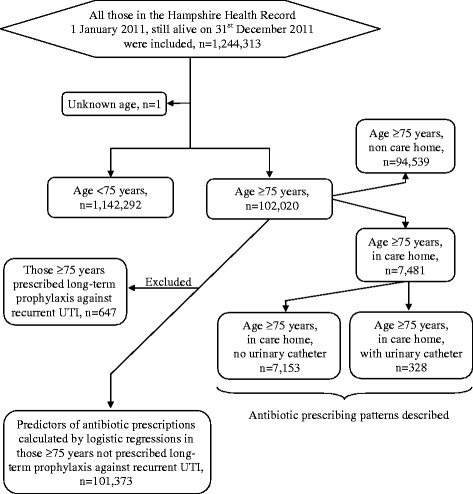
Participant flow chart

The HHR contained records of 1,244,313 residents in the Hampshire area at 1 January 2011, still alive 31 December 2011, mean age was 40 years (SD 23) and 50 % were women. 102,020 (8.2 %) were aged ≥75 years of whom 7481 (7.3 %) were presumed residents in care home settings (Table 1 and Fig. 1). In care home settings 328 (4.4 %) had urinary catheters, 5486 (73 %) were women and mean age was 87 years (SD 6.6) (Table 1). Of those in care home settings 51 % lived in a residential care home, 31 % in a nursing home and 18 % in a dual setting.

**Table 1:** Individual characteristics and antibiotic prescriptions

	Whole HHR^a^ cohort	Age <75 years	Age ≥75, non-care home	Age ≥75, in care home	Age ≥75, in care home, no urinary catheter	Age ≥75, in care home, with urinary catheter
	(N = 1,244,313)	(N = 1,142,292)	(N = 94,539)	(N = 7481)	(N = 7153)	(N = 328)
Individual characteristics						
Female	624,482 (50 %)	564,538 (49 %)	54,457 (58 %)	5486 (73 %)	5335 (75 %)	151 (46 %)
Mean age (SD)	40 (23)	36 (20)	82 (5.4)	87 (6.6)	87 (6.6)	86 (5.8)
Urinary catheter	3736 (0.30 %)	2020 (0.18 %)	1388 (1.5 %)	328 (4.4 %)	0 (0 %)	328 (100 %)
Comorbidity (QOF)^b^						
None	696,661 (56 %)	678,184 (59 %)	17,211 (18 %)	1265 (17 %)	1244 (17 %)	21 (6.4 %)
1	257,926 (21 %)	239,526 (21 %)	17,505 (19 %)	895 (12 %)	855 (12 %)	40 (12 %)
2	141,233 (11 %)	120,788 (11 %)	19,113 (20 %)	1332 (18 %)	1263 (18 %)	69 (21 %)
3 or more	148,493 (12 %)	103,794 (9.0 %)	40,710 (43 %)	3989 (53 %)	3791 (53 %)	198 (60 %)
Individuals prescribed antibiotics (AB) at least once during 2011						
Any AB^c^	329,291 (26 %)	287,067 (25 %)	38,550 (41 %)	3674 (49 %)	3405 (48 %)	269 (82 %)
UTI AB^d^	58,073 (4.7 %)	45,626 (4.0 %)	10,542 (11 %)	1905 (25 %)	1706 (24 %)	199 (61 %)
Non UTI AB^e^	301,536 (24 %)	264,466 (23 %)	34,139 (36 %)	2931 (39 %)	2717 (38 %)	214 (65 %)
Mean antibiotic prescriptions per 100 inhabitants (SD) during 2011						
Any AB^c^	61 (171)	53 (146)	142 (325)	199 (401)	188 (388)	440 (567)
UTI AB^d^	8 (560)	6 (440)	24 (116)	69 (222)	63 (209)	191 (394)
Non UTI AB^e^	53 (154)	47 (134)	118 (290)	130 (311)	125 (306)	248 (391)

^a^Hampshire Health Record (HHR)

^b^Data on comorbidity using Quality and Outcomes Framework (QOF) register coding, a system for the performance management of general practitioners in the National Health Service in the UK

^c^All antibiotics except the antiseptic substance methenamine hippurate

^d^Trimethoprim and nitrofurantoin, the dominant antibiotics used for urinary tract infection in the UK and rarely used for alternative conditions

^e^All other antibiotics than trimethoprim and nitrofurantoin

### Any antibiotic prescriptions

In the whole cohort 26 % (329,291) were prescribed antibiotics at least once during 2011 and 12 % (149,968) were prescribed antibiotics more than once. Among all ages, the median number of antibiotic prescriptions was 0 (IQR 0, 1) and the mean annual antibiotic episodes were 61 antibiotic prescriptions per 100 inhabitants (SD 171). The annual antibiotic episodes increased from 53/100 among those <75 years to 142/100 among those ≥75 years not in a care home setting and to 199/100 among those ≥75 years in a care home setting. Residents with indwelling urinary catheters in care homes had even higher use at 440/100 versus 188/100 if no urinary catheter (Table 1).

### UTI antibiotic prescriptions

The annual UTI antibiotic episodes showed a similar but more rapidly increasing pattern; 6/100 among those <75 years, 24/100 among those ≥75 years not in care home settings and 69/100 among those ≥75 years in care home settings. Residents with indwelling urinary catheters in care homes had even higher use of UTI antibiotics at 191/100 versus 63/100 if no urinary catheter (Table 1).

### Predictors of antibiotic prescriptions

Logistic regressions were performed in those ≥75 years not prescribed long-term prophylaxis against recurrent UTI. This excluded 647/102,020 individuals ≥75 years (0.63 %) prescribed long-term prophylaxis against recurrent UTI of whom 84 also had a urinary catheter (Fig. 1).

Those in care homes were more likely to have been prescribed UTI antibiotics (nitrofurantoin or trimethoprim) (Table 2), but there was a significant interaction between being in a care home and having a urinary catheter for UTI antibiotics, OR 0.52, 95 % (0.40–0.68; *p* < 0.001). The adjusted OR for UTI antibiotic prescription in nursing home residents was 2.2 (2.1–2.3; *p* < 0.001) for people without a catheter and 1.4 (1.1–1.8; *p* < 0.012) for people with an indwelling urinary catheter (Table 2).

**Table 2:** Predictors of UTI^a^ antibiotic prescriptions among those ≥ 75 years^b^

	All ≥ 75 years		≥75 years without a catheter	≥ 75 years with a catheter
	Unadjusted odds ratio	Adjusted odds ratio^c^	Adjusted odds ratio^c^	Adjusted odds ratio^c^
Care home resident	2.66 (2.51–2.82; *p* < 0.001)	2.12 (1.99–2.26; *p* < 0.001)	2.19 (2.05–2.34; *p* < 0.001)	1.40 (1.08–1.82; *p* = 0.012)
Female	2.57 (2.45–2.69; *p* < 0.001)	2.85 (2.72–2.99; *p* < 0.001)	2.95 (2.80–3.09; *p* < 0.001)	1.46 (1.16–1.84; *p* = 0.001)
Age	1.03 (1.03–1.04; *p* < 0.001)	1.01 (1.00–1.01; *p* = 0.003)	1.01 (1.00–1.01; *p* = 0.004)	0.99 (0.98–1.01; *p* = 0.574)
Urinary catheter	8.08 (7.31–8.93; *p* < 0.001)	11.3 (10.1–12.6; *p* < 0.001)	N/A	N/A
Comorbidity				
• None	1.00	1.00	1.00	1.00
• One	1.65 (1.53–1.79; *p* < 0.001)	1.67 (1.54–1.81; *p* < 0.001)	1.71 (1.58–1.86; *p* < 0.001)	0.86 (0.56–1.31; *p* = 0.475)
• Two	2.12 (1.97–2.28; *p* < 0.001)	2.04 (1.89–2.20; *p* < 0.001)	2.10 (1.94–2.27; *p* < 0.001)	0.96 (0.65–1.42; *p* = 0.832)
• Three or more	2.82 (2.64–3.02; *p* < 0.001)	2.61 (2.44–2.80; *p* < 0.001)	2.71 (2.53–2.91; *p* < 0.001)	1.00 (0.71–1.42; *p* = 0.989)

^a^Trimethoprim and nitrofurantoin, the dominant antibiotics used for urinary tract infection in the UK and rarely used for alternative conditions

^b^All ≥75 years not prescribed long term prophylaxis against recurrent UTI, both in care homes and not in care homes (*N* = 101,373)

^c^Care home resident status adjusted for gender, age, presence of urinary catheter and number of comorbidity according to Quality and Outcomes Framework, OR (95 % CI; *p*-value)

The other significant predictors of having at least one prescription of UTI antibiotics were female sex, presence of urinary catheter, comorbidity and age (Table 2).

Those in care homes were more likely to have been prescribed any antibiotic, adjusted odds ratio 1.2 (1.2–1.3; *p* < 0.001), after controlling for age, gender, urinary catheters and comorbidity (Table 3).

**Table 3:** Predictors of prescriptions of any antibiotics^a^ among those ≥75 years^b^

	Unadjusted odds ratio	Adjusted odds ratio^c^
	(95 % CI; *p*-value)	(95 % CI; *p*-value)
Care home resident	1.37 (1.31–1.44; *p* < 0.001)	1.24 (1.18–1.30; *p* < 0.001)
Female	1.27 (1.24–1.31; *p* < 0.001)	1.29 (1.25–1.32; *p* < 0.001)
Age	1.01 (1.01–1.01; *p* < 0.001)	1.00 (0.99–1.00; *p* = 0.849)
Urinary catheter	4.78 (4.27–5.37; *p* < 0.001)	4.72 (4.20–5.32; *p* < 0.001)
Comorbidity		
• None	1.00	1.00
• One	1.83 (1.75–1.92; *p* < 0.001)	1.82 (1.74–1.91; *p* < 0.001)
• Two	2.33 (2.23–2.43; *p* < 0.001)	2.28 (2.18–2.39; *p* < 0.001)
• Three or more	3.33 (3.20–3.46; *p* < 0.001)	3.22 (3.10–3.35; *p* < 0.001)

^a^All antibiotics except the antiseptic substance methenamine hippurate

^b^All ≥75 years not prescribed long term prophylaxis against recurrent UTI, both in care homes and not in care homes (*N* = 101,373)

^c^Care home resident status adjusted for gender, age, presence of urinary catheter and number of comorbidity according to Quality and Outcomes Framework

## Discussion

### Summary

In those ≥75 years, residence in a care home setting is associated with high rates of consumption of antibiotics, especially UTI antibiotics in those with an indwelling urinary catheter. However care home residency per se increases the risk of UTI antibiotic use more in people without catheters, and the effect of care home residency is present but less for any antibiotics.

### Strengths and limitations

Most previous studies covering large European care home populations are point prevalence studies collecting data from single days [17–22]. In single day point prevalence studies it is only possible to calculate what proportion of residents was using antibiotics on the day of the survey. A long-course therapy is more likely to be ongoing at a single assessment day than a short-course antibiotic therapy. Thus it is also difficult to interpret the distribution between different kinds of antibiotics in point prevalence studies and for a short term acute condition, period prevalence gives a more valid assessment of the problem.

In contrast this study includes routine data from a whole year from a large population of all ages hence countering the influence of the substantial seasonal relationship for antibiotic prescriptions [15]. By including routine data on elderly residents both in care homes and not in care homes, this study could evaluate the significance of living in a care home as well as other predictors of antibiotic prescriptions in this group. This study also allowed comparison of antibiotic prescriptions for younger and elderly inhabitants.

Large variations in antibiotic use have been noted in different care homes [17, 23]. As we extracted data from 7481 residents in care home settings, our study is expected to describe a reliable average of the use of antibiotics in UK care home settings.

There are several limitations resulting from using routine data. In the HHR, clinical coding associated with care home residency was very low and could not be used in the analysis. Instead residents of nursing and residential homes were identified by postcode. In urban areas more than one house could be sharing the same postcode, thus there could be a few people not being a resident of a care home, included in the care home cohort. To minimise this only people aged ≥75 years were included in the care home cohort. An audit was undertaken to verify this assumption using practice registered lists of five practices in different geographical locations (three urban two rural). The sample included 49 postal codes with 812 residents, of those aged 75 years and over 23/370 (6.2 %) were not care or nursing home residents. The inclusion of some non-care home residents is likely to lead to an underestimate of antibiotic use among residents at care homes, this strengthens the conclusion that residence in a care home setting is associated with high antibiotic consumption.

When the data collection was carried out it was not possible to extract date of death, so we could only identify survivors of the study period. Therefore, those in their last year of life were not included (i.e., who died during the year of study). It may be that this group would experience high antibiotic consumption.

Clinical coding of diagnoses associated with antibiotic prescriptions was very low and could not be used in the analysis. Trimethoprim and nitrofurantoin are almost exclusively prescribed for UTI, thus UTI was the assumed indication for these prescriptions. Quinolones, cephalosporins and co-amoxiclav are also prescribed for other indications than UTI. In the nursing home cohort there were 1905 prescriptions of trimethoprim and nitrofurantoin but only 298 prescriptions of quionolones and even fewer prescriptions of cephalosporins and co-amoxiclav. We chose to limit “UTI antibiotics” to trimethoprim and nitrofurantoin as they dominate in number and are almost purely used for UTI. As consequence the total number of UTI antibiotics was underestimated in this study.

Data on number of comorbidities using QOF probably underestimates frailty. Some of the excess antibiotic prescribing in the care home population may be explained by co-morbidity and frailty not included in the model.

The HHR contained routine data from 60 % of the practices in Hampshire in southern England. There was no difference between practices which were versus those which were not part of the HHR in terms of demographics or geographical location/spread.

### Comparison with existing literature

Residence in a care home setting was associated with higher UTI antibiotic consumption compared to elderly not in nursing homes in a Swedish point prevalence study [18]. A substantial proportion of elderly residents were on antibiotic treatment in European point prevalence studies of antibiotic use, collecting data from one or two single days [17–22]. Antibiotic therapy was also common in point prevalence studies in Canada [24] and Norway [25] as well as in a three-month survey of 58 nursing homes in Sweden 2003 [26], and a six-month survey of 73 nursing homes in the U.S. in 2001/2002 [27]. In a Norwegian study, elderly residents of 10 nursing homes were prescribed 218 antibiotic courses per 100 residents during a twelve-month follow-up in 2007/2008 [28] which is on par with 199 annual prescriptions per 100 residents ≥75 years in care home settings in our study. The findings in our study are consistent with and strengthen these earlier works due to the large number of care home residents and extracting data from a whole year. Our study also contributes with new knowledge about the great increase of antibiotic use, especially UTI antibiotics among residents in care home settings compared to elderly not in care homes and compared to younger individuals.

### Implications for research and practice

In this study residence in care home settings is associated with high UTI antibiotic consumption. It is likely that a substantial proportion of these UTI antibiotics are of doubtful value due to a paucity of evidence-based guidelines for the management of UTI in an older population. It is therefore important to develop such guidelines, especially when considering an aging population. Improving antimicrobial stewardship is an international priority due to the evolving threat of antibiotic resistance. Limiting any unnecessary use of indwelling urinary catheters may also be important as presence of an indwelling urinary catheter is associated with high antibiotic consumption in this study.

## Conclusions

Elderly people are prescribed substantially more antibiotics than younger people. Residence in a care home setting is associated with higher risks of antibiotic consumption especially for UTIs, absolute rates are highest in those with an indwelling urinary catheter. It is important to develop evidence-based guidelines for the management of UTI in older people as well as limiting any unnecessary use of indwelling urinary catheters.
